# Alexithymia, Inner Thinking Patterns, and Perceptions of Mental Health Therapy Strategies Among Autistic Adults

**DOI:** 10.1007/s10803-024-06643-5

**Published:** 2024-11-16

**Authors:** Micah O. Mazurek, Jessica Pappagianopoulos, Sophie Brunt, Michelle Menezes, Jessica V. Smith, Mya Howard

**Affiliations:** 1 Department of Human Services, School of Education and Human Development, University of Virginia, 417 Emmet Street South, PO Box 400267, Charlottesville, VA 22904 USA; 2 Child Study Center, Yale School of Medicine, New Haven, CT USA

**Keywords:** Autism, Mental health, Inner speech, Self-talk, Visual thinking, Therapy

## Abstract

**Purpose:**

Autistic adults are at high risk for mental health challenges, yet there has been limited research on mental health interventions for this population. Individual differences in how thoughts and emotions are perceived may directly relate to the success of specific therapy strategies. This study examined whether alexithymia and inner thinking patterns relate to helpfulness and ease of use of mental health therapy strategies among autistic adults.

**Method:**

Participants (*n* = 269 autistic adults, ages 21–77) completed questionnaires assessing alexithymia, inner thinking patterns (i.e., self-talk, verbal thinking, visual thinking), and experiences with mental health therapy strategies. Ordinal logistic regressions were used to examine associations between alexithymia, inner thinking, and perceived helpfulness and ease of use of therapy strategies.

**Results:**

Autistic adults with greater alexithymia found cognitive strategies more difficult to use, while those with greater frequency of self-talk found them easier to use. By contrast, autistic adults with greater visual thinking found guided imagery strategies easier to use. There were no associations between alexithymia or inner thinking and perceived helpfulness or ease of use of behavioral strategies (exposure, behavioral activation), mind–body relaxation strategies (deep breathing, progressive muscle relaxation), or mindfulness meditation.

**Conclusions:**

The findings suggest that some mental health strategies may be more difficult to implement for some autistic adults, depending on individual thinking profiles. However, alexithymia and inner thinking patterns were unrelated to the perceived helpfulness of mental health strategies. Overall, this highlights the importance of providing individualized supports and accommodations to optimize mental health therapy for autistic adults.

Autistic adults represent a growing population in need of mental health services. As a group, they are at disproportionate risk for mental health problems, which can have serious and life-threatening consequences (Croen et al., 2015; Jokiranta-Olkoniemi et al., 2021; Lai et al., 2019; Longo et al., 2024; Rydzewska et al., 2021). Depression and anxiety are the most common and impairing of these co-conditions (Hollocks et al., 2019; Uljarević et al., 2019), contributing to functional impairment, reduced quality of life, and increased risk for suicidality (Cassidy et al., 2018; Farley et al., 2009; Gillberg et al., 2016; Hedley & Uljarević, 2018; Thiel et al., 2024). Despite the urgency of the issue, many autistic adults are not receiving appropriate outpatient mental health services (Anderson & Butt, 2018; Camm-Crosbie et al., 2019; Tint & Weiss, 2018), leading to higher rates of mental health-related emergencies and hospitalizations (Tromans et al., 2018; Vohra et al., 2017; Weiss et al., 2018). One reason for these mental health disparities may be the lack of evidence regarding which interventions will work best for autistic adults.

Although the topic has received increasing attention over the past several years (Benevides et al., 2020; Linden et al., 2023; Maddox & Gaus, 2019; Menezes et al., 2022; Spain et al., 2015; White et al., 2018), the evidence on mental health interventions for autistic adults remains very limited. A recent systematic review and meta-analysis found that the relatively few studies to date have been characterized by significant methodological limitations, including low study quality, small sample size, and high risk of bias (Linden et al., 2023). Notwithstanding these study limitations, cognitive-behavioral therapy (CBT) and mindfulness-based therapy (MBT) appear to have the most support for treatment of anxiety and depression in autistic adults to date (Benevides et al., 2020; Linden et al., 2023). Results suggest that both types of therapy may be effective for some autistic clients, yet effect sizes are generally small to medium across studies. This may be due, in part, to heterogeneity of treatment effects and individual differences that influence the relative success of specific treatment strategies. However, previous research has not yet examined individual predictors of response to specific treatment strategies among autistic adults.

A consideration of the nature of the therapeutic techniques involved in CBT and MBT may offer insight into factors that impact their effects. As multi-component treatments, both of these approaches combine separate strategies that are aimed at reducing anxiety and improving mood (Hayes & Hofmann, 2017). However, their component strategies vary greatly in the degree to which they require monitoring and discussing thoughts and feelings. This is an especially important consideration for autistic adults, who may experience unique differences in thinking and perception that are directly related to these requirements (Rozenkrantz et al., 2021; Stark et al., 2021; Williams et al., 2023). In fact, the results of a recent mixed-methods study of autistic adults’ mental health experiences found that many participants found it difficult to identify and talk about thoughts and feelings during therapy sessions (Mazurek et al., 2023). As such, the extent to which treatment strategies require this ability may affect their relative success among autistic adults.

The wide variation in demands for monitoring and discussing internal experiences is most evident across CBT components, which include both cognitive and behavioral strategies. Cognitive strategies involve helping the individual identify, evaluate, and correct inaccurate or maladaptive thoughts or beliefs. Also known as cognitive restructuring, these strategies typically require the client to monitor their “automatic” thoughts and feelings in specific situations, evaluate the evidence and accuracy of these thoughts, and replace inaccurate or unhelpful thoughts with more rational or helpful thoughts (Beck & Beck, 2020; Clark, 2013; Ezawa & Hollon, 2023). By their very nature, cognitive strategies require an ability to recognize and verbally describe inner thinking patterns. By contrast, behavioral strategies are less internally-focused and more action-oriented, with a focus on helping clients change patterns of behavior and learn new skills (Beck & Beck, 2020). For example, exposure strategies reduce anxiety through repeated engagement with feared situations over time (Abramowitz et al., 2019), while behavioral activation strategies reduce depression through scheduled engagement in pleasant or rewarding activities (Lejuez et al., 2001; Lewinsohn & Graf, 1973).

Additional skill-building strategies that are focused on increasing relaxation and reducing physiological arousal are also commonly included in CBT approaches. These also vary greatly in their demands for monitoring or modifying internal experiences or thoughts. The most common of these are deep breathing (Chen et al., 2017) and progressive muscle relaxation (Bernstein & Borkovec, 1973), both of which are bodily-focused exercises that induce a physiological relaxation response. By contrast, guided imagery is a much more internally-focused strategy requiring the individual to imagine and visualize a calm, pleasant, or relaxing scene (Cohen & Twemlow, 1981; Daake & Gueldner, 1989).

Finally, mindfulness meditation, a primary MBT strategy, is focused on developing non-judgmental awareness and acceptance of present moment experiences (Baer, 2003). Within this framework, the focus is on simply observing the ongoing stream of moment-to-moment thoughts, sensations, and emotions, without judging or trying to change them. This requires the ability to focus attention and observe internal experiences but does not involve intentional tracking or modification of thoughts or feelings.

Given the nature of these various treatment strategies, the extent to which internal experiences are easily described and the nature of how they are perceived may affect their relative success among autistic adults. Alexithymia represents an especially important aspect of inner experience for many autistic adults. Alexithymia is characterized by difficulties with identifying and describing feelings (Sifneos, 1973), and is considered to be a continuous, dimensional trait that varies across individuals (Parker et al., 2008). Autistic adults are more likely to experience difficulties with alexithymia than non-autistic adults, with approximately 50% demonstrating significant alexithymia (as compared to 5% of the general population) (Kinnaird et al., 2019). Alexithymia may relate to the core diagnostic features of autism, including difficulties with social communication and social-emotional reciprocity (American Psychiatric Association, 2013), although prior evidence suggests that it is a separable trait from autism itself (Bird & Cook, 2013; Cook et al., 2013; Oakley et al., 2020; Trevisan et al., 2016).

Alexithymia may be directly relevant to treatment response, particularly for strategies that rely on emotional monitoring and discussion. In the general population, alexithymia has been found to be a predictor of poor response to supportive and psychodynamic therapies (Leweke et al., 2009; McCallum et al., 2003; Ogrodniczuk et al., 2011). However, findings have been mixed across CBT studies, with some studies finding no association with treatment response (Kishon et al., 2020; McGillivray et al., 2019; Rufer et al., 2004, 2010), and other studies finding that alexithymia predicted worse response to CBT (Brady et al., 2015; Quilty et al., 2017). Because these studies ranged considerably in their assessment of alexithymia and CBT approach, it is possible that alexithymia may have a differential effect, depending on the specific strategies emphasized. Further research is warranted to explore how alexithymia may affect the success of specific component treatment strategies, particularly among autistic adults, who are at high risk for significant alexithymia (Kinnaird et al., 2019).

Individual differences in other aspects of inner experience may also be relevant to therapy experiences for autistic adults. For example, the extent to which thoughts are easily described (or experienced as inner speech) may be directly relevant to the success of cognitive strategies, which require monitoring and discussing thoughts. By the same token, experiencing thoughts as visual images may be relevant to the success of strategies like guided imagery, which require internal visualization of scenes. There is wide variability in the general population, and among autistic adults, in extent to which thoughts are experienced verbally or visually (Bled et al., 2021; Heavey & Hurlburt, 2008; Heavey et al., 2019). In the general population, some people experience thoughts primarily as inner speech, while others experience thoughts primarily as visual images (Heavey & Hurlburt, 2008; Hurlburt et al., 2021). Similarly, some autistic people experience thoughts visually, consistent with Temple Grandin’s experiences as described in her *Thinking in Pictures* memoir (2006), while others rely on verbal thinking strategies (Kunda & Goel, 2011; Whitehouse et al., 2006; Williams et al., 2016). As such, individual differences in these thinking patterns may represent important and treatment-relevant characteristics for autistic adults. While previous studies have examined the *content* of self-talk as it relates to mental health symptoms in non-autistic adults (Buschmann et al., 2018; Şoflău & David, 2017), the relationship between verbal or visual inner thinking modalities and the utility of mental health therapy strategies has not been previously investigated in either autistic or non-autistic populations. Because mental health strategies vary in the extent to which they require monitoring or modification of verbal or visual thoughts, understanding how individual differences in thinking patterns may relate to the relative success of these strategies and may be important for treatment planning.

## Current Study

The goal of this study was to examine whether alexithymia and inner thinking patterns relate to perceived helpfulness and ease of use of therapy strategies among autistic adults. Given that this was the first study to investigate this topic, our approach was largely exploratory. However, based on the inherent nature of some therapy strategies, as described above, we hypothesized that: 1) greater alexithymia would be associated with reduced helpfulness and ease of use of cognitive strategies, 2) greater frequency of self-talk and inner speech would be associated with increased helpfulness and ease of use of cognitive strategies, and 3) greater frequency of inner seeing/visual thinking would be associated with increased helpfulness and ease of use of guided imagery strategies.

## Method

### Participants and Procedures

Participants in the current study were recruited as part of a larger study on mental health among autistic adults (Mazurek et al., 2023). The study was approved by the Institutional Review Board at the University of Virginia and all participants provided informed consent prior to participating. Eligibility included being an autistic adult aged 21 years or older, not having a legal guardian, and having a previous professional diagnosis of autism. Recruitment was conducted through the Simons Foundation Powering Autism Research for Knowledge (SPARK) Research Match process. SPARK is a large-scale study of autistic individuals and their family members across the United States who are part of an autism research community and agree to be contacted for future research opportunities. To be eligible for SPARK enrollment, participants are required to have a previous professional diagnosis of autism spectrum disorder (ASD) and be residents of the United States. Previous research has provided strong evidence of validity of professional diagnoses within the SPARK cohort, with 98.8% of ASD diagnoses confirmed in a recent diagnostic validation study (Fombonne et al., 2022). SPARK participants enroll online, complete questionnaires, and provide saliva samples for genetic analysis. Eligible participants are then connected with additional research opportunities through the SPARK Research Match process. After the current study was approved by both the IRB and by the SPARK Participant Access Committee, the SPARK Research Match team sent invitation emails to eligible participants with basic information about the study and how to join. Participants who indicated interest then received a link to a Study Information Page, which provided more detailed information and an electronic consent form. Out of 2221 eligible participants who received an invitation email from the SPARK team, 493 indicated initial interest in the study, 339 provided consent, and 303 went on to complete the surveys for the larger study. Out of the 303 participants who completed surveys for the larger study, 269 reported previous therapy experience and were included in the current study. Participants completed all measures online and received $15 for participation.

### Measures

#### Demographic Survey

Demographic characteristics were assessed using a survey created for the study. Items included age, sex, gender, race, ethnicity, socioeconomic status, relationship status, living arrangements, and employment/student status.

#### Mental Health Therapy Experiences Survey

Mental health therapy experiences were assessed using a survey developed for the study. Items focused on prior mental health and therapy or counseling experiences. Participants with prior therapy experience were asked whether their therapy included the following specific therapeutic strategies: cognitive strategies, mindfulness meditation, deep breathing, guided imagery, progressive muscle relaxation, exposure, and behavioral activation. If their previous therapy had included a specific strategy, follow-up questions were: “How helpful were these strategies?” (1 = *not at all helpful* to 5 = *very helpful*), and “How easy were these strategies to use? (1 = *very difficult* to 5 = *very easy*). Participants were asked to consider all previous therapy or counseling experiences and to focus on their “most recent experience” with each type of strategy (if applicable). To ensure that participants were able to accurately identify specific strategies, each item began with the question: “Did your therapy include [specific strategy]?” and included a more detailed definition of that term. The definitions were as follows: “**Cognitive strategies** include paying attention to your thoughts, identifying unhelpful thoughts, challenging or reframing negative thoughts, and developing more balanced and helpful thoughts. **Mindfulness meditation** involves focusing on the present moment, becoming aware of sensations and thoughts, and observing and allowing them to pass without judging them. **Deep breathing** focuses on learning to take slow, deep breaths. **Guided imagery** or **visualization** focuses on imagining a scene that is peaceful and relaxing to you. **Progressive muscle relaxation** involves slowly tensing and relaxing your muscle groups one at a time. **Exposure** involves gradually facing anxiety-provoking situations until they no longer create anxiety or fear. **Behavioral activation** involves scheduling and increasing positive and enjoyable daily activities to improve your mood.”

#### Depression and Anxiety Symptoms

Symptoms of anxiety and depression were assessed using the Patient Health Questionnaire Anxiety-Depression Scale (PHQ-ADS) (Spitzer et al., 1999), a 16-item questionnaire assessing current symptoms of both depression (9 items, PHQ-9) and anxiety (7 items, GAD-7). Items are rated on a 4-point scale (1 = *not at all* to 4 = *nearly every day*), with higher scores indicating greater symptom severity. The PHQ-ADS is well-validated and has strong psychometric properties in the general population (Kroenke et al., 2010, 2019; Spitzer et al., 1999) and in autistic adults (Arnold et al., 2019; Russell et al., 2020; Syu et al., 2020).

#### Alexithymia

The Toronto Alexithymia Scale (TAS-20) was administered to assess alexithymia. The TAS-20 is a widely used measure of alexithymia with strong psychometric properties in the general population (Bagby et al., 1994, 2020) and in autistic individuals (Kinnaird et al., 2019). Items are rated on a 5-point scale ranging from *Strongly Disagree* to *Strongly Agree*. Items are summed to create a total score, with higher scores indicating increased alexithymia, and a score of ≥ 61 indicating clinically relevant alexithymia.

#### Self-Talk

Frequency of self-talk was assessed using the Self-Talk Scale (STS; Brinthaupt et al., 2009). The STS is a 16-item measure of the frequency and functions of inner speech. Items are rated on a 5-point frequency scale (*Never* to *Very Often*) and are summed to create a total score, with higher scores indicating greater frequency of self-talk. The STS has strong psychometric properties in the general population (Brinthaupt & Kang, 2014; Brinthaupt et al., 2009, 2015). Cronbach’s alpha was 0.937 in the current sample of autistic adults, demonstrating strong internal consistency.

#### Inner Speaking and Inner Seeing

The relative frequency of inner speaking (i.e., verbal thinking) and inner seeing (i.e., visual thinking) was assessed using the Nevada Inner Experience Questionnaire (NIEQ) (Heavey et al., 2019). The NIEQ is a self-report measure assessing different types of inner experiences with strong psychometric properties (Heavey et al., 2019). Each type of experience is assessed with a pair of items, which are rated on a visual analog scale ranging from 0 to 100. Each item pair is summed to generate subscale domain scores. For the current study, the Inner Speaking and Inner Seeing subscales were examined. Inner Speaking items include: “How frequently do you talk to yourself in your inner voice?” (*Never* to *Always*) and “Generally speaking, what portion of your inner experience is in inner speech (thinking in words)?” (*None* to *All).* Inner Seeing items include: “How frequently do you mentally see or visualize something?” (*Never* to *Always*) and “Generally speaking, what portion of your inner experience is in images (seeing things in your imagination)?” (*None* to *All).*

### Data Analysis Plan

Descriptive statistics were used to characterize key variables, including mean, standard deviation, range, and percentage. Before testing primary hypotheses, initial analyses were conducted to determine whether demographic variables were related to perceived helpfulness or ease of use of mental health therapy strategies. Due to the ordinal nature of the mental health strategy variables, non-parametric methods were used, including Spearman-rank correlations (for age and highest level of education), Mann–Whitney U tests (for ethnicity), and Kruskal–Wallis H tests (for race, gender identity, and highest level of education).

Bivariate correlations were first conducted between inner experience variables (alexithymia, self-talk, inner speaking, and inner seeing) and helpfulness or ease of use of each mental health strategy (cognitive strategies, mindfulness meditation, deep breathing, guided imagery, progressive muscle relaxation, exposure, and behavioral activation). Spearman-rank correlations were selected due to the ordinal nature of the mental health strategy variables. To reduce the possibility of Type I error, a Bonferroni correction was applied, dividing the original alpha level of *p* = 0.05 by the total number of analyses per inner experience variable (i.e., 14). Using this correction, the adjusted significance level was set at *p*_corrected_ < 0.003.

After ensuring all assumptions were met, a series of cumulative odds ordinal logistic regressions with proportional odds were conducted to examine the relative contribution of each type of inner experience to either the helpfulness or ease of use of each mental health therapy strategy. Separate analyses were conducted for each type of strategy: cognitive strategies, mindfulness meditation, deep breathing, guided imagery, progressive muscle relaxation, exposure, and behavioral activation. Total PHQ-ADS score was included as a covariate in each model to control for the effects of mental health symptom severity.

## Results

Participants ranged in age from 21 to 77 years (*M* = 37.5, *SD* = 12.1), and were largely White (85.8%), non-Hispanic/Latino (94.8%). Regarding sexual orientation and gender identity, most participants were straight (57.6%) and most identified as cis-gender women (51.3%). When asked about autism terminology preferences, 42.2% preferred identity-first language (“autistic person”), 28.9% preferred “person on the autism spectrum,” 16.0% preferred “person with autism,” and 12.9% preferred other terms. In terms of timing of autism diagnosis, the average age of diagnosis was 26.7 (*SD* = 15.3), and the majority (59.9%) were diagnosed in adulthood (at or after age 21). Most participants had a history of at least one co-occurring mental health diagnosis, including: anxiety disorder (80.3% lifetime, 63.6% current), depression (81.8% lifetime, 53.2% current), attention-deficit/hyperactivity disorder (ADHD; 54.6% lifetime, 40.9% current), post-traumatic stress disorder (PTSD; 46.1% lifetime, 34.9% current), obsessive compulsive disorder (OCD; 24.2% lifetime, 14.9% current), and bipolar disorder (21.6% lifetime, 9.7% current). See Table 1 for additional sample characteristics.

**Table 1:** Sample Characteristics (*n* = 269)

	Number (Percent)
Gender Identity	
Cisgender woman	138 (51.3%)
Cisgender man	76 (28.3%)
Transgender woman	6 (2.2%)
Transgender man	10 (3.7%)
Non-binary/non-conforming	34 (12.6%)
Other	5 (1.9%)
Sexual Orientation	
Straight	155 (57.6%)
Gay or lesbian	20 (7.4%)
Bisexual or pansexual	59 (21.2%)
Asexual	22 (8.2%)
Other	15 (5.6%)
Ethnicity	
Hispanic/Latino	14 (5.2%)
Race	
American Indian or Alaska Native	1 (0.4%)
Asian	3 (1.1%)
Black or African American	13 (4.8%)
White or Caucasian	230 (85.5%)
Multiracial	21 (7.8%)
Residence	
Live with romantic partner or spouse	102 (37.9%)
Live alone	73 (27.1%)
Live with parent(s)	67 (24.9%)
Live with roommate(s)	27 (10.0%)
Other	29 (10.8%)
Level of Education	
Less than high school	5 (1.9%)
High school graduate or GED	70 (26.0%)
Associate or technical degree	38 (14.1%)
Bachelor’s degree	95 (35.3%)
Master’s degree	51 (19.0%
Doctoral degree	10 (3.7%)
Employment	
Full time job	105 (39.0%)
Part-time job, student	12 (4.5%)
Part-time job, not student	40 (14.9%)
Not employed, student	21 (7.8%)
Not employed, not student	91 (33.8%)

Of the total sample of autistic adults with a history of previous therapy experience (*n* = 269), the frequency and percentage who had received any of these specific mental health strategies was as follows: cognitive strategies (*n* = 201, 74.7%), mindfulness meditation (*n* = 121, 45.0%), deep breathing (*n* = 144, 53.5%), guided imagery (*n* = 83, 30.9%), progressive muscle relaxation (*n* = 57, 21.2%), exposure (*n* = 53, 19.7%), and behavioral activation (*n* = 75, 27.9%). Descriptive statistics for the inner experience variables were as follows: TAS-20 (*M* = 59.7, *SD* = 13.1), Self-Talk Scale (*M* = 58.6, *SD* = 13.8), NIEQ Inner Speaking (*M* = 63.7, *SD* = 24.3), NIEQ Inner Seeing (*M* = 53.9, *SD* = 29.0).

Except for a small correlation between age and ease of use of deep breathing strategies (*r* = -0.169, *p* = 0.044), there were no statistically significant relationships between any other demographic factors and either helpfulness or ease of use of mental health strategies. Bivariate correlations between inner experience patterns and helpfulness and ease of use of mental health therapy strategies are shown in Table 2. There were no statistically significant correlations between variables after correcting for multiple comparisons (effect sizes were generally small, ranging from* r* = − 0.008 to *r* = 0.327).

**Table 2:** Bivariate correlations between inner experiences and perceptions of therapy strategies

	Alexithymia^a^	Self-talk^b^	Inner speaking^c^	Inner seeing^d^
Cognitive Strategies (*n* = 201)				
Helpfulness	− .116	.039	.039	.146
Ease of Use	− .213	.087	− .005	.070
Mindfulness Meditation (*n* = 121)				
Helpfulness	− .188	.043	.076	.105
Ease of Use	− .051	− .023	.041	.065
Deep Breathing (*n* = 144)				
Helpfulness	− .092	.110	− .018	.151
Ease of Use	− .070	.059	.013	.062
Guided Imagery (*n* = 83)				
Helpfulness	− .003	.026	− .024	.250
Ease of Use	− .035	.112	.102	.327
Progressive Muscle Relaxation (*n* = 57)				
Helpfulness	− .193	.083	− .131	.159
Ease of Use	− .210	− .008	.009	− .091
Exposure (*n* = 53)				
Helpfulness	− .136	.191	− .152	.090
Ease of Use	− .258	− .112	− .136	− .149
Behavioral Activation (*n* = 75)				
Helpfulness	− .113	.179	.053	− .055
Ease of Use	− .225	− .005	− .060	.084

None of the correlations were statistically significant after Bonferroni correction

*p*_corrected_ < 0.003

^a^Toronto Alexithymia Scale (TAS-20) total score

^b^Self-Talk Scale total score

^c^Nevada Inner Experience Questionnaire (NIEQ) Inner Speaking subscale score

^d^NIEQ Inner Seeing subscale score

### Multivariate Relations Among Inner Experiences and Mental Health Therapy Strategies

#### Cognitive Strategies

The ordinal logistic regression model examining predictors of helpfulness of cognitive strategies (*n* = 199) found that none of the inner experience variables were significant predictors. However, an increase in PHQ-ADS score was associated with a decrease in the odds of finding cognitive strategies helpful, with an odds ratio of 0.966, 95% CI [0.943, 0.989], χ^2^(1) = 8.158, *p* = 0.004. The model predicting ease of use of cognitive strategies (*n* = 199) found that both alexithymia and frequency of self-talk were statistically significant predictors. An increase in Self-Talk Scale score was associated with an increase in the odds of finding cognitive strategies easy to use, with an odds ratio of 1.023, 95% CI [1.000, 1.046], χ^2^(1) = 3.971, *p* = 0.046; while an increase in TAS-20 score was associated with a decrease in the odds of finding cognitive strategies easy to use, with an odds ratio of 0.969, 95% CI [0.950, 0.990], χ^2^(1) = 8.694,* p* = 0.003.

#### Mindfulness Meditation

The ordinal logistic regression models examining predictors of helpfulness and ease of use of mindfulness meditation strategies (*n* = 120) were not a good fit to the observed data; χ^2^(5) = 6.693, *p* = 0.244, and χ^2^(5) = 1.729, *p* = 0.885, respectively.

#### Deep Breathing Strategies

The ordinal logistic regression models examining predictors of helpfulness and ease of use of deep breathing strategies (*n* = 140) were not a good fit to the observed data; χ^2^(5) = 5.785, *p* = 0.328, and χ^2^(5) = 1.636, *p* = 0.897, respectively.

#### Guided Imagery Strategies

The ordinal logistic regression model examining predictors of helpfulness of guided imagery (*n* = 82) was not a good fit to the observed data; χ^2^(5) = 8.646, *p* = 0.124. However, the model examining predictors of ease of use of guided imagery/visualization found that inner seeing was a significant predictor. An increase in NIEQ Inner Seeing subscale score was associated with an increase in the odds of finding guided imagery easy to use, with an odds ratio of 1.024, 95% CI [1.009, 1.040], χ^2^(1) = 9.377, *p* = 0.002.

#### Progressive Muscle Relaxation

The ordinal logistic regression models examining predictors of helpfulness and ease of use of progressive muscle relaxation (*n* = 56) were not a good fit to the observed data; χ^2^(5) = 5.821, *p* = 0.324, and χ^2^(5) = 2.684, *p* = 0.748, respectively.

#### Exposure

The ordinal logistic regression models examining predictors of helpfulness and ease of use of exposure (*n* = 51) were not a good fit to the observed data; χ^2^(5) = 6.222, *p* = 0.285, and χ^2^(5) = 8.182, *p* = 0.146, respectively.

#### Behavioral Activation

The ordinal logistic regression models examining predictors of helpfulness and ease of use of behavioral activation (*n* = 73) were not a good fit to the observed data; χ^2^(5) = 6.901, *p* = 0.228, and χ^2^(5) = 6.658, *p* = 0.247, respectively.

## Discussion

This was the first study to examine the relationship between inner thinking patterns and mental health therapy strategies among autistic adults. The results offer new insights into personalized approaches to treatment planning for autistic adults with co-occurring mental health conditions. Autistic adults experience extremely high rates of depression, anxiety, and other mental health concerns (Fombonne et al., 2020; Jadav & Bal, 2022; Lai et al., 2019; Lugo-Marín et al., 2019). As such, optimizing the effectiveness of mental health treatment for autistic adults is of utmost importance. The results of this study provide some interesting insights into the usability of specific strategies based on individual differences in how thoughts and emotions are experienced.

Consistent with our predictions, inner thinking patterns were most closely related to the usability of therapeutic strategies that rely on monitoring or manipulating internal thoughts or images. Specifically, autistic adults with greater alexithymia found cognitive strategies more difficult to use, while those who engaged in more frequent self-talk found cognitive strategies easier to use. This finding makes sense in terms of the inherent demands of cognitive therapy strategies. For example, these approaches typically require clients to track, record, and discuss “automatic thoughts” and associated feelings as they occur throughout the day or in specific situations. For autistic adults with significant alexithymia, identifying thoughts in the context of strong emotions may be challenging, due to their difficulties reflecting on and verbally describing emotional processes (Taylor & Bagby, 2004). Similarly, for individuals who rarely engage in self-talk, it may be more difficult for them to identify, label, and monitor automatic thoughts; whereas this may be much easier and more relevant for those who engage in a high frequency of self-talk.

Interestingly, contrary to our hypothesis, the degree to which individuals experience thoughts as inner speech was not related to the usability of cognitive strategies. This may be due to a slight difference in the constructs assessed by the Self-Talk Scale as compared to the NIEQ Inner Speaking subscale. The Self-Talk Scale assesses not only the frequency of self-talk (how often people talk to themselves silently or aloud) across various situations, but also on the functional context (e.g., self-criticism, self-reinforcement, self-management, and social assessment) (Brinthaupt et al., 2009). By contrast, the NIEQ Inner Speaking subscale simply measures the relative frequency of talking to oneself in an inner voice (thinking in words, rather than other modalities) (Heavey et al., 2019). It is possible that these additional contextual aspects of thought captured by the Self-Talk Scale may be particularly relevant to treatment, above and beyond the idea of “verbal thinking” alone. For example, cognitive therapy typically focuses on thoughts that are triggered by specific social or environmental contexts rather than moment-to-moment spontaneous thoughts.

While alexithymia and self-talk were related to ease of use of cognitive strategies, none of the inner experience variables were related to perceived helpfulness of these strategies. This lack of relationship was surprising and contrary to our predictions. These findings indicate that while some inner thinking patterns may make cognitive strategies more difficult for some people to learn, this may not relate to their overall perceived effectiveness. It may also be the case that cognitive strategies actually improve alexithymia and/or increase awareness of internal thinking, especially in emotionally charged situations. In fact, one mechanism by which cognitive therapy is thought to achieve its effect is through increasing metacognition (Dobson, 2013) [i.e., recognizing and understanding one’s own cognitive processes, (Flavell, 1979)]. Essentially, cognitive strategies help individuals develop greater awareness of their own thoughts and feelings by learning to observe, evaluate, and restructure their own emotion-related thought processes (Clark, 2013; Ezawa & Hollon, 2023). From this perspective, even for individuals who have initial difficulty with these skills, actively learning and practicing within a therapeutic context may be beneficial. Together, these results suggest that autistic adults with alexithymia and those who rarely engage in self-talk may still benefit from cognitive strategies, but that they may need additional support to learn and practice them.

Also consistent with our predictions, guided imagery strategies were easier to use for autistic adults who were more likely to experience thoughts as images (i.e., visual thinking). This is not surprising, given that guided imagery strategies require an individual to imagine a visual scene. For those who visualize thoughts more readily, these strategies may simply be easier to implement. Surprisingly, however, there was not a relationship between visual thinking and perceived helpfulness of guided imagery strategies. It may be the case that the relaxation response achieved through these strategies may not be directly related to the vividness of the imagined scene, but to other factors. Consistent with this idea, a study of non-autistic adults found that the ability to generate mental images was unrelated to the success of guided imagery strategies, but that the capacity to become *absorbed* in imagination was predictive of success (Kwekkeboom et al., 1998). This suggests that additional aspects of the inner experience, including absorption, may be helpful for understanding treatment fit for autistic adults.

In terms of other mental health strategies, the findings suggest that alexithymia and inner thinking patterns are not significantly related to either the perceived helpfulness or ease of use of behavioral strategies (i.e., exposure or behavioral activation), mind–body relaxation strategies (i.e., deep breathing, progressive muscle relaxation), or mindfulness meditation. Because this was the first study on the topic, we had no empirical or theoretical basis for making directional predictions for these variables. However, it is interesting to note that the ease of use and perceived effectiveness of these more action-oriented strategies appear unrelated to the way in which autistic adults perceive and describe their thoughts and emotions. Overall, this suggests that such strategies may be equally acceptable across the population regardless of thinking style, and/or that their effectiveness depends on other personal or clinical characteristics.

## Limitations and Future Directions

This study was largely exploratory, as it was the first to examine the relationship between inner thinking patterns and mental health therapy experiences among autistic adults. As such, there are several limitations that are important to note. First, the study was limited to autistic adults who were independent (i.e., did not have legal guardians) and who had the ability to read and respond to written survey questions. As such, the results may not be generalizable to those with significant cognitive, language, or literacy difficulties, although it should be noted that the study did not include specific measures of cognitive functioning, language, or level of independence. Further assessment of these domains may be informative to evaluate the extent to which these skills relate to inner thinking patterns and mental health strategies. Additionally, due to the nature of the online study, we were unable to conduct independent diagnostic assessments to confirm previous professional diagnoses of autism.

The sample was also limited in terms of racial and ethnic diversity and may not reflect the experiences of the broader population of autistic adults. It is also worth noting that a greater proportion of participants in the current sample identified as female as compared to the broader autism population (Maenner et al., 2023). However, the gender ratio in the current sample may reflect the population of autistic adults seeking mental health therapy services in a real-world context, given robust evidence that women are much more likely than men to seek mental health services (Addis & Mahalik, 2003; Judd et al., 2008; Parent et al., 2018; Seidler et al., 2016), including those with disabilities (Manning et al., 2023).

Another notable limitation is that the current study relied on self-reported retrospective recall of both inner thinking and mental health therapy experiences. In addition, the survey did not assess the timing or duration of participants’ therapy experiences, nor how long ago they were received. As such, responses may have been affected by recall bias. A more direct and comprehensive assessment of inner experience patterns may be useful in future research, including qualitative, mixed-methods, and ecological momentary assessment techniques across settings. It should also be noted that the current analyses were correlational in nature, meaning that causality or directionality cannot be determined. A prospective approach is needed in future studies to examine causality and to explore the relations among these variables over time. Robust study designs, including randomized clinical trials, would be useful to examine the comparative effectiveness of specific mental health strategies as well as moderators and mediators of treatment response. Within such designs, individual characteristics (including alexithymia and inner thinking) could be examined as potential predictors, moderators, mediators, and/or outcomes of mental health treatment strategies. Finally, it should be noted that not all participants had received each specific mental health strategy. As a result, the sample size varied across analyses examining the relations among inner experiences and helpfulness and ease of use of each strategy. This differentially affected our power to detect statistically significant associations between variables across these models. Future research with larger samples would be helpful to continue to explore these issues.

## Conclusions

This study provides the first examination of how alexithymia and inner thinking patterns might relate to autistic adults’ perceptions of mental health strategies. The findings suggest that some strategies may be more difficult to implement for some autistic adults, depending on how they experience and describe their inner thinking and feelings. However, there were no associations between these inner experiences and the ultimate helpfulness of mental health strategies. Overall, treatment acceptability likely depends on the fit between the demands of the strategy and the characteristics of the autistic client. This highlights the importance of individualized accommodations and a personalized approach for mental health treatment for autistic adults. Future research is needed to evaluate individual predictors, mediators, and moderators of treatment response in this population. This knowledge will be vital for optimizing usability and effectiveness of mental health treatments for autistic adults.
